# Diversity of Epigenetic Features of the Inactive X-Chromosome in NK Cells, Dendritic Cells, and Macrophages

**DOI:** 10.3389/fimmu.2018.03087

**Published:** 2019-01-08

**Authors:** Camille M. Syrett, Vishal Sindhava, Isabel Sierra, Aimee H. Dubin, Michael Atchison, Montserrat C. Anguera

**Affiliations:** Department of Biomedical Sciences, School of Veterinary Medicine, University of Pennsylvania, Philadelphia, PA, United States

**Keywords:** Xist RNA, X-chromosome inactivation, long non-coding RNA, plasmacytoid dendritic cells, macrophages, sex differences, NK cells, interferon alpha

## Abstract

In females, the long non-coding RNA Xist drives X-chromosome Inactivation (XCI) to equalize X-linked gene dosage between sexes. Unlike other somatic cells, dynamic regulation of Xist RNA and heterochromatin marks on the inactive X (Xi) in female lymphocytes results in biallelic expression of some X-linked genes, including *Tlr7, Cxcr3*, and *Cd40l*, implicated in sex-biased autoimmune diseases. We now find that while Xist RNA is dispersed across the nucleus in NK cells and dendritic cells (DCs) and partially co-localizes with H3K27me3 in bone marrow-derived macrophages, it is virtually absent in plasmacytoid DCs (p-DCs). Moreover, H3K27me3 foci are present in only 10–20% of cells and we observed biallelic expression of *Tlr7* in p-DCs from wildtype mice and NZB/W F1 mice. Unlike in humans, mouse p-DCs do not exhibit sex differences with interferon alpha production, and interferon signature gene expression in p-DCs is similar between males and females. Despite the absence of Xist RNA from the Xi, female p-DCs maintain dosage compensation of six immunity-related X-linked genes. Thus, immune cells use diverse mechanisms to maintain XCI which could contribute to sex-linked autoimmune diseases.

## Introduction

In the immune system, long non-coding RNAs (lncRNAs) are being increasingly recognized as important regulators of gene expression for both innate and adaptive immune responses (1). Indeed, lncRNAs can function as regulators of immune cell differentiation, lymphocyte activation, and inflammatory responses. For example, the lncRNA Morrbid is abundantly expressed in nuclei of neutrophils, eosinophils, and monocytes, and Morrbid deletion reduces the numbers of these short-lived myeloid cells (2). Similar to Morrbid, lnc-DC is also upregulated during differentiation of common myeloid progenitors into dendritic cells (DCs), and regulates DC differentiation through cytoplasmic interactions with the transcription factor STAT3 (3). Activation of DCs and macrophages through specific TLRs results in dramatic upregulation of lincRNA-Cox2, which regulates over 500 genes encoding inflammatory molecules (4).

One of the best characterized lncRNAs is Xist, which is required for silencing the X-chromosome during X-chromosome Inactivation (XCI). Females use XCI for dosage compensation of X-linked genes between the sexes. XCI is initiated during early female mammalian embryonic development (5) by allele-specific upregulation of Xist from the future inactive X (Xi) (6–8). Xist RNA functions in *cis* to recruit chromatin complexes that deposit heterochromatic modifications (including H3K27me3 and H2a-ubiquitin) across the X, resulting in transcriptional silencing (9–11). During XCI maintenance, these epigenetic modifications are enriched on the Xi and contribute to its transcriptional silencing after cell division, to ensure dosage compensation of X-linked genes. In differentiating embryonic stem cells, *Xist* is continuously expressed from the Xi throughout the cell cycle, and Xist RNA remains tethered to the Xi of its origin throughout mitosis (12).

The majority of somatic cells maintain XCI through continuous expression of *Xist* from the Xi, and enrichment of Xist RNA transcripts and heterochromatin marks on the Xi are cytologically visible. Surprisingly, we have shown that mature naive T and B cells from female mice and humans lack these epigenetic modifications on the Xi. However, Xist RNA and some heterochromatin modifications are present on the Xi in *in vitro* activated lymphocytes (13, 14), suggesting that XCI is dynamically regulated in lymphocytes. Using RNA FISH, Xist RNA localization patterns in lymphocytes can be categorized into four classes: Type I Xist RNA patterns exhibit robust signals, Type II patterns have dispersed signals within the X-chromosome territory, Type III patterns have diffuse signals across the nucleus, and Type IV patterns lack detectible signal (14, 15). This dynamic localization of Xist RNA and heterochromatin marks suggests relaxed transcriptional silencing on the Xi, which is supported by recent observations by our group and others of biallelic expression of the X-linked genes *Tlr7, Cxcr3*, and *Cd40l* in mouse and human T and B cells (14, 16).

Based on our findings in lymphocytes, we assessed Xist RNA localization patterns on the Xi in terminally differentiated myeloid and lymphoid-derived cells. We found that NK cells and dendritic cells (DCs) have Xist RNA transcripts dispersed across the nucleus, while bone marrow derived macrophages (BMDMs) have Xist RNA pinpoints clustered at the Xi, and exhibit co-localization of Xist RNA and the heterochromatin mark H3K27me3. Interestingly, resting and activated plasmacytoid DCs (p-DCs) lack Xist RNA localization at the Xi, and most cells also lack H3K27me3. Additionally, we observed biallelic expression of *Tlr7* in wildtype and disease-stage NZB/W F1 p-DCs, yet there were no sex differences with interferon alpha production, unlike in human cells. Together, these data reveal that immune cells use diverse mechanisms to maintain XCI that could contribute to sex-linked autoimmune diseases.

## Materials and Methods

### Mice

Female mice (aged 2–6 months) of various backgrounds (C57BL/6, BALB/c, NZB × NZW F1) were purchased from Jackson Laboratories, and used to isolate bone marrow derived macrophages (BMDM), NK cells, dendritic cells (DCs), and plasmacytoid DCs. All mice were maintained at the Penn Vet animal facility. Animal experiments were approved by the University of Pennsylvania Institutional Animal Care and Use Committee (IACUC). Euthanasia via carbon dioxide was used for animal sacrifice prior to spleen isolation.

### Fluorescence Activated Cell Sorting (FACS) Isolation of NK Cells, Lymphoid and Myeloid Dendritic Cells From Spleen

Spleens were harvested on ice in FACS buffer (PBS/3%FCS) and single-cell suspensions were prepared by meshing cells through 40-um strainers, then cells were stained with antibodies for fluorescence activated cell sorting (FACS) analyses. Briefly, cells were stained with fluorochrome-conjugated or biotinylated antibodies to mouse. Staining was performed in PBS/1%BSA containing mouse IgG Fc fragments (Jackson Immunoresearch, Cat # 115-006-020). Dead cells and doublets were excluded and sorting was performed on a FACS Aria II machine using the following markers at a concentration of 1:100 unless otherwise specified: NK cells: TCRb+CD19 (H57-597/6D5, BioLegend), NK1.1 (PK138, BD Pharmingen), NKP46 (29A1.4, eBiosciences). m-DCs: CD11c (N418, BioLegend), CD11b (M1/70, eBiosciences, 1:200). L-DCs: CD8a (53-6.7, eBiosciences). Data were analyzed using FlowJo software.

### Isolation and *in vitro* Stimulation of Plasmacytoid Dendritic Cells (p-DCs) and Bone Marrow Derived Macrophages

Plasmacytoid dendritic cells (p-DCs) were isolated from spleen and peripheral lymph nodes by negative selection using a plasmacytoid dendritic cell isolation kit (#130-107-093, Miltenyi Biotec). p-DCs were cultured in RPMI-1640 containing 2 mM L-glutamine, 10% FCS, 1% Pen/Strep and 50 μM β-mercaptoethanol. P-DCs were stimulated with 1 μM CpG (ODN 1826, InvivoGen) and cultured for 3 days.

Bone marrow was isolated from female 6 week old C57BL/6J mice and cultured in complete DMEM (10% FBS, 1% NaPyruvate, 1% HEPES, 30% L929 conditioned medium) and re-fed on day 4. Macrophages were isolated 8 days after differentiation by washing petri culture dishes with Mg^2+^ and Ca^2+^ EDTA-free 4C PBS. Under these culture conditions we estimate that the population of BMDMs is 98% pure using flow cytometry (data not shown). Cells were re-plated with complete DMEM with 10% L929 conditioned media and stimulated with either 1 μM CpG (ODN 1826, InvivoGen) or 1 μg/mL LPS (Sigma) for 3 days.

### Xist RNA FISH, Tlr7 RNA FISH, and Immunofluorescence Detection of H3K27me3

Sequential RNA fluorescence *in situ* hybridization (FISH) and immunofluorescence (IF) for immune cells was performed following established protocols for splenocytes (14, 15), where Xist RNA FISH was performed first followed by IF for the same locations on the slides. For Xist RNA FISH, two Cy3-labeled 20-nucleotide oligo probes were designed to recognize regions within Xist RNA exon 1 (synthesized by IDT). For IF, cells were blocked with 0.2% PBS-Tween, 0.5% BSA. Histone H3K27me3 (Active Motif; Cat. #39155) was diluted 1:100 for IF. Single-molecule RNA FISH for Tlr7 was performed according to Stellaris protocols, using Cy3-labeled oligo probes for exonic regions (Stellaris), and FITC-labeled oligo probes for intronic regions (Stellaris). Images were obtained using a Nikon Eclipse microscope and were categorized by the four types of Xist RNA localization patterns as described previously (14, 15). Statistical significance was calculated using two-tailed *t*-tests and ANOVA.

### Analysis of Gene Expression and IFN-α Protein Production in p-DCs

To determine levels of X-lined and IFNα gene expression, p-DCs were isolated from spleens of (NZB × NZW) F1 [NZB/W F1] mice by negative selection using a plasmacytoid dendritic cell isolation kit (#130-107-093, Miltenyi Biotec). p-DCs were cultured as described above and stimulated with 10 μg/mL R848 (Resiquimod, Sigma Aldrich) or 1 μM CpG (InvivoGen) and cultured for 6 h. Cells and supernatants were collected after 6 h of culturing (NS: unstimulated; R848, CPG: activated). RNA was isolated using TRIzol (Invitrogen), and cDNA was synthesized with qScript cDNA SuperMix (Quanta). qRT-PCR was performed using the following primer pairs [For Seq 5′-3′; Rev Seq 5′-3′]: Rpl13a [AGC CTA CCA GAA AGT TTG CTTAC; GCT TCT TCT TCC GAT AGT GCA TC], Xist [GCT GGT TCG TCT ATC TTG TGGG; CAG AGT AGC GAG GAC TTGA AGAG], Cxcr3 [TAC CTT GAG GTT AGT GAA CGT CA; CGC TCT CGT TTT CCC CAT AATC], Cfp [TTC ACC CAG TAT GAG GAG TCC; GCTG ACC ATT GTG GAG ACCT], Irak1 [TCC TCC ACC AAG CAG TCA AG; AAA ACC ACC CTC TCC AAT CCT], Il2rg [CTC AGG CAA CCA ACC TCAC; GCT GGA CAA CAA ATG TCT GGT AG], Msn [GGCT TCC CGT GGA GTG AAA TC; GTC CGG GGC CTT TTT GTC AA], Tlr7 [ATG TGG ACA CGG AAG AGA CAA; GGT AAG GGT AAG ATT GGT GGTG], Ifna2 [TAC TCA GCA GACC TTG AAC CT; CAG TCT TGG CAG CAA GTT GAC], Ccl4 [TTC CTG CTG TTT CTC TTA CACCT; CTG TCT GCC TCT TTT GGT CAG], Irf7 [CTC CTG AGC GCA GCC TTG; GTT CTTAC TGC TGG GGC CAT], Ifit2 [GGA GAG CAA TCT GCG ACAG; GCT GCC TCA TTT AGA CCT CTG].

To determine expression levels, the housekeeping gene Rpl13a was used for normalization (2^∧^ΔΔCT). Combined qRT-PCR results are shown from three independent experiments.

Total serum IFNα from *in vitro* p-DCs cell culture supernatants was measured using a VeriKine Mouse IFN alpha ELISA Kit (42120, pbl assay science). Supernatants were collected after 6 h of culture and were undiluted for ELISA. The plate was read at 450 nm immediately after development and was analyzed using protein standards provided in the kit (400, 200, 100, 50, 25, 12.5, 0 pg/mL).

## Results

### NK Cells Predominantly Lack Xist RNA on the Xi and Xist RNA Is Dispersed Across the Nucleus in Dendritic Cells

**Figure 1:**
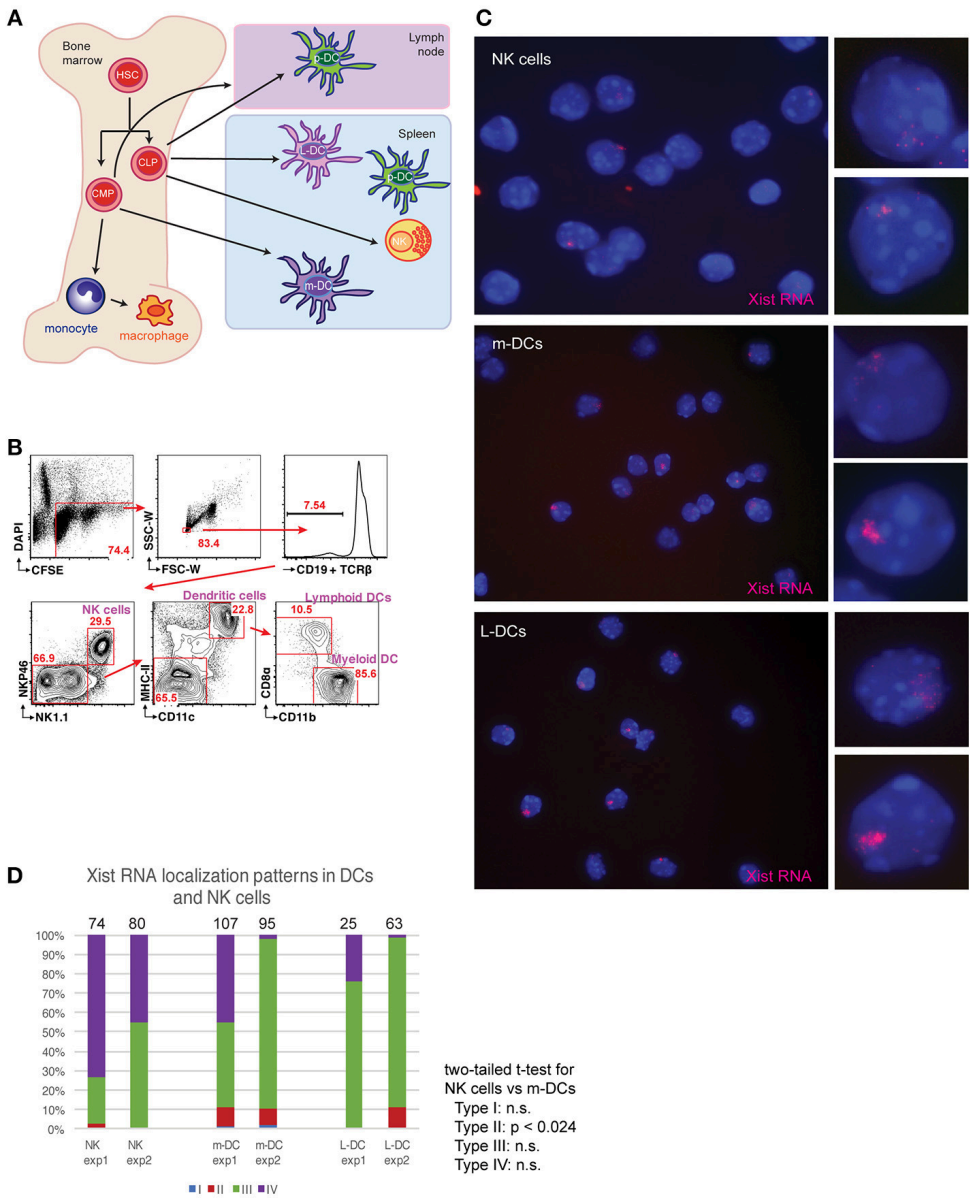
Xist RNA transcripts are mostly absent from the Xi in female NK cells and DCs. **(A)** Schematic showing the origin for the immune cells examined here. Hematopoietic stem cells (HSCs); common lymphoid progenitors (CLPs); common myeloid progenitors (CMPs); plasmacytoid dendritic cells (p-DCs); myeloid-derived DCs (m-DCs); lymphoid-derived DCs (L-DCs). **(B)** Sorting strategy for isolation of m-DCs (MHC-II^+^, CD11c^+^, CD11b^+^, CD8a^lo^), L-DCs (MHC-II^+^, CD11c^+^, CD11b^+^, CD8a^hi^), and NK cells (NK1.1^+^) using FACS. Spleens from two female mice were pooled for each experiment (repeated twice), and flow results from experiment 1 are shown. **(C)** Xist RNA FISH analyses of NK cells, m-DCs, L-DCs, using Cy3 labeled oligo probes. **(D)** Quantification of Xist RNA localization patterns (Types I–IV) for each experiment. The total number of nuclei counted for each cell type is shown above the column. Statistical significance was determined comparing each type of Xist RNA pattern (Types I–IV) for each cell type, using a two-tailed *t*-test. The comparison between NK cells and m-DCs for Type II patterns was the only significant difference (*p* < 0.024). NK cells and L-DCs had no significant differences in Xist RNA localization patterns.

To determine if XCI is dynamically regulated in NK cells, lymphoid-DCs (L-DCs), and myeloid DCs (m-DCs) each of these cell types were isolated from female mouse spleens (Figure 1A). These cells are derived from common lymphoid progenitors and common myeloid progenitors, which reside in the bone marrow, and are known to have robust Xist RNA “clouds” on the Xi (15). We used fluorescence activated cell sorting (FACS) to isolate each population following the surface marker profiling shown in Figure 1B, then immediately cytospun and fixed the cells on glass slides, which preserves nuclear RNA signals. We used Cy3-labeled short oligo probes for Xist to perform RNA FISH, and classified the percentage of cells for each localization pattern (Types I-IV) (14, 15). NK cells predominantly lacked detectible Xist RNA signals (Type IV) and 20–50% of cells exhibited Type III patterns with diffuse Xist RNA pinpoints dispersed across the nucleus (Figures 1C,D). M-DCs and L-DCs had about 10% Type II cells, where Xist RNA pinpoints are localized in a nuclear territory encompassing the inactive X (Xi), and 40–90% Type III cells (Figure 1D). These results show that NK cells have less Xist RNA localized to the Xi compared to m-DCs and L-DCs, and suggest that NK cells may have more genes that escape XCI than DCs.

### Xist RNA and H3K27me3 Modifications Are Localized to the Xi in Bone Marrow Derived Macrophages (BMDM)

**Figure 2:**
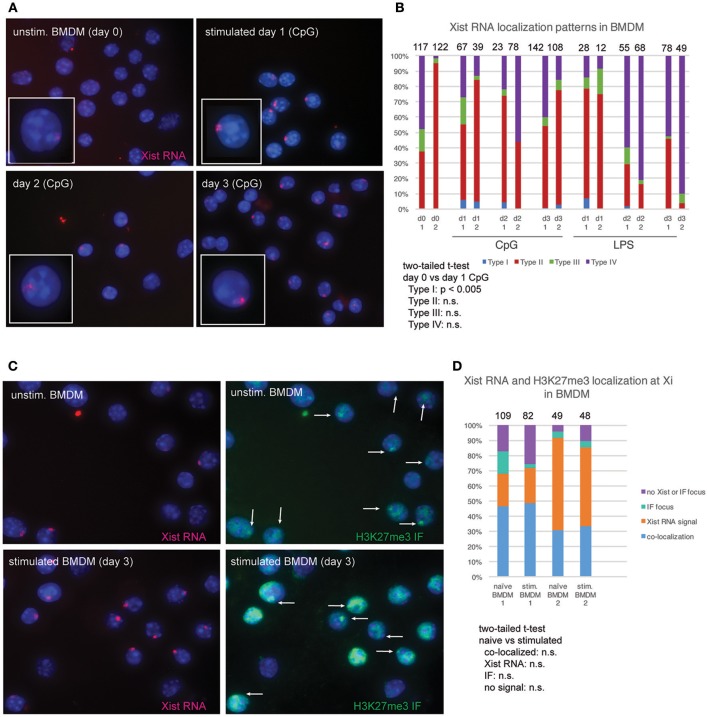
Xist RNA and H3K27me3 foci are localized to the Xi in most female BMDMs. **(A)** Xist RNA FISH for resting BMDMs and *in vitro* stimulated cells (using 1 μM CpG), collected 3 days after stimulation. **(B)** Quantification of Xist RNA localization patterns for BMDMs stimulated with either 1 μM CpG or 1 μg/mL LPS. The total number of nuclei counted for each cell type is shown above the column. Statistical significance for comparisons of resting (day 0) vs. stimulated cells was performed for each type of Xist RNA localization pattern (Types I–IV) using a two-tailed *t*-test, and the only significant difference was for CpG-stimulated Type I cells (*p* < 0.005). **(C)** Sequential Xist RNA and H3K27me3 IF for resting and stimulated BMDMs. White arrows indicate H3K27me3 foci. **(D)** Quantification of co-localization patterns for Xist RNA and H3K27me3 foci. Results from two independent experiments are shown. The total number of nuclei counted for each cell type is shown above the column. Statistical significance for comparisons of resting (day 0) vs. stimulated cells was performed for each type of localization pattern using a two-tailed *t*-test, and the only significant difference was for CpG-stimulated Type I cells (*p* < 0.005).

Cytokine production and phagocytic activity of macrophages exhibits sex-related differences (17, 18). As the expression of X-linked genes could contribute to these functional differences, we asked whether Xist RNA and H3K27me3 are localized to the Xi in macrophages. We cultured BMDMs for 8 days after isolation, and then activated the cells for 3 days using CpG or LPS. Unstimulated BMDMs had mostly Type II Xist RNA patterns (40–90%) and some Type III (~5–10%) (Figures 2A,B). Stimulation with either CpG or LPS increased the number of Type I Xist RNA patterns for about 5–10% of cells, yet the percentage of Type II and Type III patterns did not significantly change (Figures 2A,B). The number of Type I cells decreased by day 3 for both CpG and LPS stimulation, and Xist RNA signal persisted longer and in more cells with CpG stimulation (Figures 2A,B). Next, we examined the co-localization of Xist RNA signals with H3K27me3 foci using sequential RNA FISH followed by IF. As shown in Figures 2C,D, Xist RNA signals co-localized with a focus of H3K27me3 in 30–50% of BMDMs, and *in vitro* stimulation did not change the level of co-localization (Figure 2C). About 20–50% of cells had an Xist RNA signal yet lacked H3K27me3 foci, and very few cells (5–12%) had a H3K27me3 focus and lacked Xist RNA signal (Figures 2C,D). These results suggest that Xist RNA localization at the Xi is necessary for H3K27me3 enrichment on this chromosome in BMDMs.

### Plasmacytoid DCs Lack Xist RNA and H3K27me3 foci on the Xi and Biallelically Express *Tlr7*

**Figure 3:**
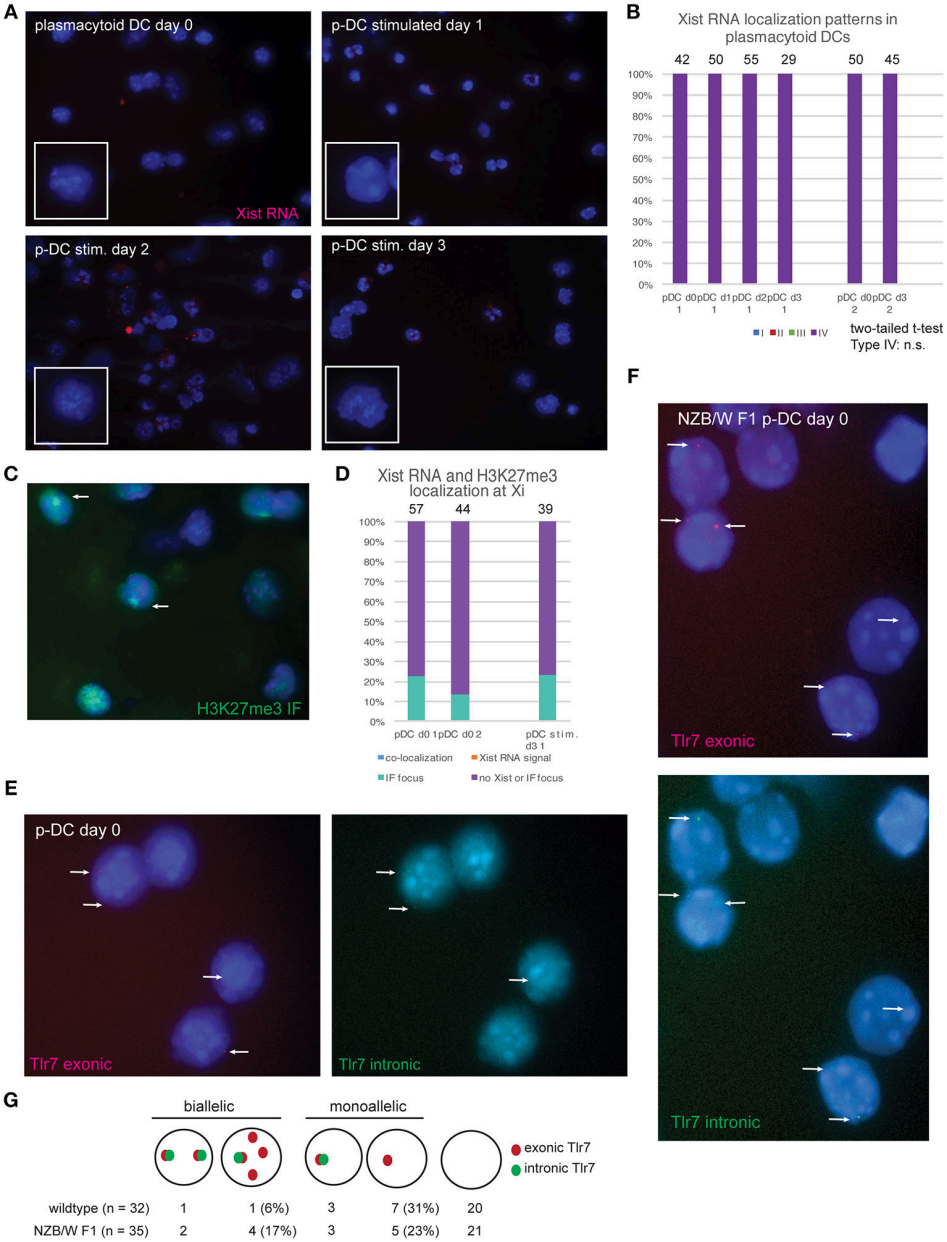
Female plasmacytoid DCs lack Xist RNA at the Xi and exhibit biallelic expression of *Tlr7* in some cells. **(A)** Xist RNA FISH for resting p-DCs and *in vitro* activated p-DCs after days 1–3 of culture. P-DCs were isolated from spleen and lymph nodes from female mice, in two independent experiments and stimulated with CpG. For the second isolation, cells were stimulated for 3 days before collection for RNA FISH. **(B)** Quantification of Xist RNA localization patterns for p-DCs showing that all p-DCs are missing Xist RNA on the Xi. The total number of nuclei counted for each cell type is shown above the column. **(C)** Sequential Xist RNA FISH followed by immunofluorescence (IF) for H3K27me3 enrichment at the Xi. **(D)** Quantification of co-localization patterns for Xist RNA and H3K27me3 foci. Results from two independent experiments are shown. The total number of nuclei counted for each cell type is shown above the column. **(E)** Single-molecule RNA FISH for Tlr7 transcripts using wildtype p-DCs from healthy mice. Oligo probes specific for exonic *Tlr7* were Cy3-labeled (red), and intronic *Tlr7* probes were FITC-labeled (green). White arrows indicate pinpoint signals for nascent *Tlr7* expression from the X-chromosome, with signals from both exonic and intronic probes. **(F)** single-molecule *Tlr7* RNA FISH in p-DCs from NZB/W F1 mice with SLE-like disease. Disease development was assessed by proteinuria and DNA autoantibodies prior to p-DC isolation from spleen and lymph nodes. White arrows indicate pinpoint signals for nascent *Tlr7* expression from the X-chromosome, with signals from both exonic and intronic probes. **(G)** Schematic for counting allele-specific expression, and quantification of monoallelic and biallelic Tlr7 expression in wildtype and NZB/W F1 p-DCs. The percentages of total numbers for biallelic and monoallelic expressing cells are shown in parentheses.

Plasmacytoid DCs (p-DCs) are a distinct lineage of DCs that produce interferon (IFN) in response to viral nucleic acids detected by TLR7 and TLR9 (19). TLR7-mediated stimulation of female plasmacytoid DCs (p-DCs) from human females results in higher levels of IFN regulatory factor 5 (IRF5) and IFNα compared to p-DCs from males (20, 21). TLR7 is an X-linked gene that is prone to escape XCI in female B and T cells (14, 16), and exhibits elevated expression in some female immune cells (22). In female Systemic Lupus Erythematosus (SLE) patients, p-DCs are a major source of aberrant IFN production that contributes to disease progression (23). We asked whether Xist RNA was localized to the Xi in p-DCs pooled from lymph nodes and spleen using RNA FISH. Surprisingly, we did not detect any Xist RNA signal in p-DCs, and 100% of the cells were Type IV (Figures 3A,B). Xist RNA signals were also absent from the Xi and the nucleus in LPS or CpG-stimulated p-DCs (Figures 3A,B). Next, we investigated whether the repressive chromatin modification H3K27me3, which localizes to the Xi in fibroblasts and some activated lymphocytes, was present in p-DCs. Using sequential RNA FISH followed by immunofluorescence (IF) detection, we found that the majority of p-DCs lacked H3K27me3 foci (Figure 3C) and that 10–20% of p-DCs had a detectible focus of H3K27me3 (Figure 3D). In sum, p-DCs lack Xist RNA localization to the Xi and enrichment of H3K27me3, suggesting that the chromatin of the Xi may be prone to reactivation of some X-linked genes.

To determine whether the absence of Xist RNA localization to the Xi affects *Tlr7* expression in p-DCs, we performed RNA FISH using oligo probes specific for the exonic and intronic regions of *Tlr7*. Resting p-DCs had low yet detectible signals for Tlr7 RNA: the majority of cells lacked Tlr7 RNA pinpoints, yet we could identify some cells with monoallelic (one pinpoint) and also biallelic (two pinpoints) expression (Figure 3E). Next, we isolated p-DCs from (NZB × NZW) F1 female mice (NZB/W F1), which is a high quality model of spontaneous SLE-like disease with a strong female bias. Disease was assessed by proteinuria, serum double strand DNA antibodies, and sudden weight loss (24). NZB/W F1 female mice have increased numbers of p-DCs and produce more IFNα compared to healthy female C57BL/6 mice (25). The p-DCs from diseased female mice had more robust exonic *Tlr7* signals and more biallelic Tlr7 expression compared to healthy mice (Figures 3F,G). Thus, the absence of Xist RNA and H3K27me3 enrichment on the Xi in p-DCs correlates with elevated expression of *Tlr7* during SLE-like disease.

### Female p-DCs do not Require Xist RNA Localized on the Xi for X-linked Gene Dosage Compensation

**Figure 4:**
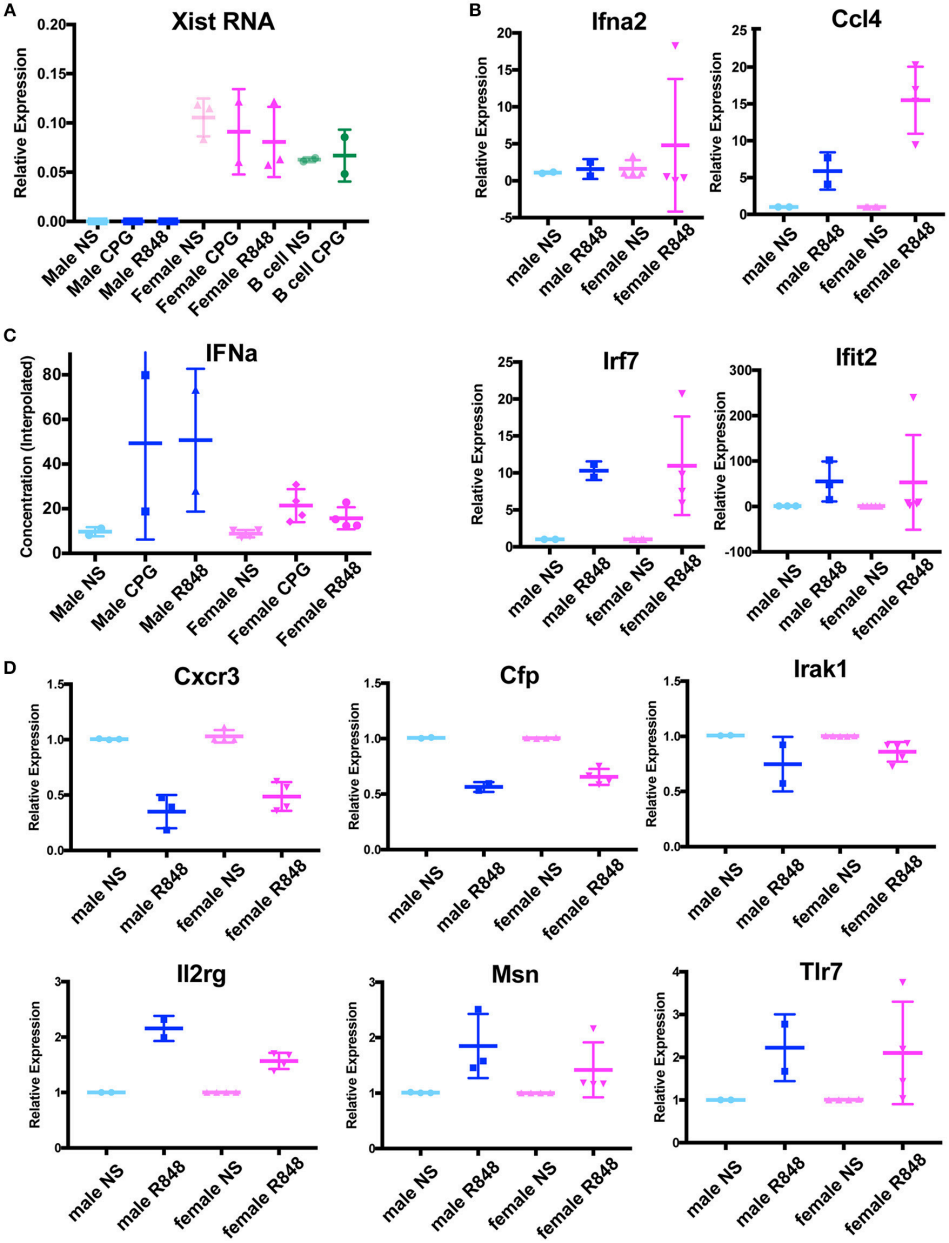
p-DCs do not exhibit a sex difference with IFNα production and X-linked genes are dosage compensated in the absence of Xist RNA localization at the Xi. **(A)** Relative quantity (2^∧^ΔCt) of Xist RNA in unstimulated male and female pDCs, and cells stimulated with CPG, R848. Female B cells (naïve and CPG stimulated) were included as positive controls. **(B)** Relative quantity (2^∧^ΔΔCt) of four IFNα signature genes. P-DCs were activated with R848 for 6 h or were unstimulated (NS). The housekeeping gene Rpl13a was used for normalization, and male unstimulated samples (NS) were normalized to 1. **(C)** Concentration of IFNα protein produced by cultured male and female p-DCs from NZB/W F1 mice measured by ELISA. **(D)** Relative quantity (2^∧^ΔΔCt) of six X-linked immune genes from male and female splenic p-DCs from NZB/W F1 mice. P-DCs were activated with R848 for 6 h or were unstimulated (NS). The housekeeping gene Rpl13a was used for normalization, and unstimulated samples (NS) were normalized to 1.

Because female p-DCs lacked Xist RNA and H3K27me3 enrichment at the Xi (Figure 3), we first asked whether Xist RNA was transcribed in these cells. We isolated splenic p-DCs from males and females, and stimulated cells using two methods (CPG or R848), then isolated total RNA for qRT-PCR. We also included naïve and stimulated B cells for comparison, as Xist RNA levels are similar between naïve B cells that lack Xist RNA localization at the Xi and stimulated cells which have robust Xist RNA clouds (15). *Xist* is expressed at similar levels in unstimulated and stimulated female pDCs, regardless of the method of activation (Figure 4A). Thus, *Xist* transcription and localization are uncoupled in pDCs, and this may account for the absence of H3K27me3 enrichment on the Xi in these cells.

We next asked if female pDCs from NZB/W F1 mice produced more of the X-linked *Tlr7* gene than males, and if this resulted in higher levels of IFNα. To determine whether p-DCs exhibited sex-biased gene expression of IFNα and IFNα signature genes, we isolated splenic p-DCs from male and female NZB/W F1 mice at early and late stage disease. We cultured the cells in the presence or absence of the Tlr7 agonist R848 for 6 h, then harvested cells for RNA isolation. We used qRT-PCR to determine the steady state levels for the IFNα signature genes *Ifna2, Ccl4, Irf7*, and *Ifit2*, which are expressed in p-DCs (26, 27). We saw no significant sex differences between the expression of these genes in female and male p-DCs stimulated with R848 (Figure 4B).

Higher levels of IFNα production have been reported in p-DCs from human females compared to males (20, 21). Next, we asked whether pDCs from NZB/W F1 mice exhibited sex differences with IFNα protein levels. To determine if female p-DCs produced more IFNα than male p-DCs, we determined the IFNα concentration in supernatants of cultured male and female p-DCs (resting and *in vitro* activated using R848) by ELISA. While the IFNα concentrations were variable, we saw no significant increase in IFNα production in female p-DCs (Figure 4C), suggesting that, unlike in humans, murine female and male p-DCs produce similar levels of IFNα.

Next, we asked whether female p-DCs, which lack Xist RNA at the Xi, exhibit greater expression of X-linked genes known to be subject to XCI. We performed qRT-PCR for six X-linked immune genes expressed in p-DCs from male and female NZB/W F1 mice. We did not observe any significant sex differences with the expression of *Cxcr3, Cfp, Irak1, Il2rg, Msn*, and *Tlr7* (Figure 4D). Together, these results suggest that female mouse p-DCs are capable of maintaining X-linked gene dosage compensation in the absence of Xist RNA localized at the Xi.

## Discussion

Taken together, our findings reveal wide diversity in the localization of the epigenetic modifications Xist RNA and H3K27me3 at the Xi in myeloid and lymphoid lineages. This new insight may have important implications for understanding how X-linked gene expression from the Xi is regulated in diverse immune cell populations. NK cells have faint and dispersed Xist RNA signals across the nucleus (Type III) and nuclei that lack Xist RNA (Type IV), which suggests that some X-linked genes in these cells may be prone to reactivation. Dosage of the X-linked gene *XIAP* affects NK cell function in patients with X-linked lymphoproliferative syndrome presenting with chronic inflammatory bowel disease (28), which underscores the importance of X-linked gene expression in NK cells. We found that resting BMDMs, unlike lymphocytes, have predominantly Type II Xist RNA patterns, and that *in vitro* stimulation with CpG generates few Type I cells. Thus, the epigenetic features of the Xi in female BMDMs more closely resembles that of female fibroblasts, but with less robust Xist RNA clouds. Xist RNA localization on the Xi is correlated with H3K27me3 foci in BMDMs, which is observed in fibroblasts (9), differentiating mouse embryonic stem cells (29), and activated B cells (15).

We also found that splenic m-DCs and L-DCs have more robust and detectible Xist RNA signals compared to NK cells, with most of these DCs classified as Type III with dispersed Xist RNA across the nucleus and some cells being Type II with clustered Xist RNA pinpoints. In contrast, p-DCs are completely distinct from other DCs, lymphocytes, and BMDMs as they lack detectible Xist RNA and are exclusively Type IV. However, we observed that 10–20% of p-DCs have H3K27me3 foci, which suggests that Xist RNA localization at the Xi is not required for H3K27me3 enrichment in these cells. It is possible that the 80–90% of p-DCs that lack Xist RNA/H3K27me3 enrichment are primed for gene-specific reactivation from the Xi. In support, we observe biallelic expression of *Tlr7* in some p-DCs from both healthy and disease-state NZB/W F1 female mice. These results support a model where Xist RNA and heterochromatin marks localized on the Xi promote transcriptional silencing, and gene reactivation may occur from the Xi more readily when these epigenetic modifications are missing.

Despite the absence of Xist RNA transcripts on the Xi, it was surprising that female mouse p-DCs maintained dosage compensation of six X-linked immune genes, including *Tlr7*. We hypothesize that the fidelity of transcriptional silencing of these genes on the Xi is likely maintained by DNA methylation and additional heterochromatin marks (besides H3K27me3) in female p-DCs. It is possible that there are X-linked genes besides the six examined here that specifically escape XCI in female mouse p-DCs. Future experiments that detect allele-specific expression from the Xi will reveal whether Xist RNA localization influences gene reactivation in p-DCs. It has been reported that human female p-DCs have elevated *TLR7* expression and increased IFNα production compared to male cells (21). We were surprised to find that male and female p-DCs from NZB/W F1 mice express similar levels of *Tlr7*, and that IFNα concentrations from *in vitro* cultured cells did not exhibit sex differences. Our findings suggest that the Xi in female pDCs is more transcriptionally silent than the human Xi in p-DCs, which is supported by the observations that the human Xi from various tissues contains more genes that escape XCI (15–25% X-linked genes escape XCI) compared to the mouse Xi (30, 31). We cannot exclude the possibility that human p-DCs may also lack Xist RNA on the Xi, which contributes to female-specific overexpression of *TLR7* in human p-DCs, and that increased expression could come from both the Xa and Xi. *TLR7* has been recently shown to escape XCI in healthy human B cells (16), and it is possible that *TLR7* might be bi-allelically expressed in human p-DCs. Additional studies examining the allelic expression profiles of X-linked genes in human p-DCs are necessary to reveal the origins of female-biased *TLR7* expression. In conclusion, our results demonstrate that female murine immune cells use diverse mechanisms to maintain XCI, which may underlie sex differences with some immune responses and the observed sex-bias in predisposition to autoimmune diseases.

## Author Contributions

CS and IS isolated the plasmacytoid DCs and BMDMs. CS performed RNA FISH and quantification of Xist RNA localization patterns. CS performed the IF, single molecule RNA FISH for *Tlr7*, and gene expression analyses. VS performed flow cytometry and cell sorting for NK cells and DCs. AD performed the ELISA. MA provided funding for flow cytometry and for VS, MCA, and CS wrote the manuscript. All authors read and approved the final manuscript.

### Conflict of Interest Statement

The authors declare that the research was conducted in the absence of any commercial or financial relationships that could be construed as a potential conflict of interest.

## Abbreviations

Xi: inactive X
XCI: X-chromosome Inactivation
DC: dendritic cell
p-DC: plasmacytoid dendritic cell
IF: immunofluorescence
BMDM: bone marrow derived macrophages
L-DC: lymphoid dendritic cell
M-DC: myeloid dendritic cell.
